# Facile Synthesis of Sandwich-Type Porous Structured Ni(OH)_2_/NCNWs/rGO Composite for High Performance Supercapacitor

**DOI:** 10.3390/molecules30051119

**Published:** 2025-02-28

**Authors:** Xiaosen Duan, Mingyu Dou, Lingyang Liu, Long Zhang, Xianrui Bai, Ruixin Yang, Hengyi Wang, Jianmin Dou

**Affiliations:** Shandong Provincial Key Laboratory of Chemical Energy Storage and Novel Cell Technology, School of Chemistry and Chemical Engineering, Liaocheng University, Liaocheng 252059, China; zssls231402@163.com (X.D.); doumingyu@lcu.edu.cn (M.D.); zglgme@163.com (L.Z.); 17852261385@163.com (X.B.); yangruixin1019@163.com (R.Y.); wanghengy19@163.com (H.W.)

**Keywords:** supercapacitor, Ni(OH)_2_, reduced graphene oxide, positive electrode, N-doped porous carbon

## Abstract

Nickel hydroxide has ultra-high energy storage capacity in supercapacitors, but poor electrical conductivity limits their further application. The use of graphene to improve its conductivity is an effective measure, but how to suppress the stacking of graphene and improve the overall performance of composite materials has become a new challenge. In this work, a well-designed substrate of N-doped carbon nanowires with reduced graphene oxide (NCNWs/rGO) was fabricated by growing polypyrrole (PPy) nanowires between GO nanosheets layers and then calcining them at high temperatures. This NCNWs/rGO substrate can effectively avoid the stacking of rGO nanosheets, and provides sufficient sites for the subsequent in situ growth of Ni(OH)_2_, forming a uniform and stable Ni(OH)_2_/NCNWs/rGO composite material. Benefiting from the abundant pores, high specific surface area (107.2 m^2^ g^−1^), and conductive network throughout the NCNWs/rGO substrate, the deposited Ni(OH)_2_ can not only realize an ultra-high loading ratio, but also exposes more active surfaces (221.3 m^2^ g^−1^). After a comprehensive electrochemical test, it was found that the Ni(OH)_2_/NCNWs/rGO positive materials have a high specific capacitance of 2016.6 F g^−1^ at a scan rate of 1 mV s^−1^, and exhibit significantly better stability. The assembled Ni(OH)_2_/NCNWs/rGO//AC asymmetric supercapacitor could achieve a high energy density of 85.2 Wh kg^−1^ at power densities of 381 W kg^−1^. In addition, the asymmetric supercapacitor has excellent stability and could retain 70.1% of initial capacitance after 10,000 cycles. These results demonstrate the feasibility of using NCNWs/rGO substrate to construct high-performance supercapacitor electrode materials, and it is also expected to be promoted in other active composite materials.

## 1. Introduction

With the rapid depletion of fossil fuels, there is a growing requirement to ameliorate a range of problems caused by energy shortages, environmental pollution, and climate change [1,2]. Therefore, research into development, transportation, and storage of green energy sources is receiving increased attention [3]. This urgently requires electrochemical energy storage devices with high energy density, high storage efficiency, and high stability. The rapid expansion of global renewable energy demand promotes the development of novel electrochemical energy storage devices with higher energy density, high storage efficiency, and high stability [4,5]. Supercapacitors, with their safety, high power density, and ultra-long cycle life, have become an effective alternative to conventional batteries in high-power-demanding applications [6]. There are two mechanisms to energy storage for supercapacitors, including electric double layer capacitance (EDLC) and pseudocapacitance. EDLC relies on surface adsorption of electrolyte ions for charge storage, such as various carbon materials, including graphene, porous carbon, carbon nanotubes (CNTs), carbon nanowires (CNWs), and graphitic carbon nitride (g-C_3_N_4_) [7,8,9,10,11]. Pseudocapacitance, meanwhile, is based on faradaic processes, such as transition metal oxides (TMOs), transition metal hydroxides (TMOHs), transition metal sulfides (TMSs), conducting polymers (CPs) and carbonitrides (MXenes) [12,13,14,15,16,17].

In recent years, there have been numerous reports on the study of the modification of carbon materials and TMOs/TMOHs and their application as electrode materials in supercapacitors. For example, graphene is regarded as an ideal electrochemical material due to its good conductivity, high specific surface area, and surface modifiability, while the bottleneck of low energy density and easy stacking restricts its specific capacitance [18]. Among TMOs/TMOHs, Ni(OH)_2_ is widely researched due to its low cost, high theoretical specific capacitance, and flake-like morphology, but its poor electrical conductivity significantly restricts the electrochemical performance and rate performance [19]. Each of the graphene and Ni(OH)_2_ materials has its own unique advantages, and when utilized in composite form, these materials exhibit enhanced properties. To avoid blockage of ion transport channels by stacking graphene sheets, Li and coworkers developed a porous graphene material by assembling graphene oxide (GO) and exfoliated graphene (EG) into EG-mediated GO (EGM-GO) films with a specific capacitance (C_sp_) of 231 F g^−1^ [20]. Liu et al. reported that through polymerization of polyaniline (PANI) onto the reduced graphene oxide (rGO) hydrogel film, they synthesized rGO/PANI composite hydrogel film electrode exhibiting a C_sp_ of 854 F g^−1^. [21]. Similarly, Liu and co-workers synthesized a composite of 3D rose-like β-Ni(OH)_2_/rGO by a one-step hydrothermal method, and this electrode showed a C_sp_ of 802 F g^−1^ [22]. Cai and coworkers reported a composites of Ni(OH)_2_/N-CNF synthesized using polypyrrole (PPy)-coated cellulose as the precursor with the C_sp_ of 1045 F g^−1^ [23]. Numerous studies have shown that using graphene as a substrate to load Ni(OH)_2_ is an effective method for preparing high-performance supercapacitor electrode materials [24,25]. The comparison of specific capacitance values of electrode materials among the mentioned literature are summarized in Table S1. It can be seen from these reports that the enhanced performance of the composites was primarily attributable to the synergistic effect between the high-specific-capacity pseudocapacitive component and the conductive substrate. If the conductive substrate provided enough available surface to realize a large loading mass, the synergistic effect between the functional components would be fully exploitable. Consequently, the construction of substrates with multilevel pores, plenty of defects, and large practical specific surface area served as a pivotal foundation for the development of high-performance electrochemical composites.

Therefore, this study first prepared a high-performance substrate material using GO and PPy, and then loaded high-performance Ni(OH)_2_ onto the substrate to obtain a ternary composite material. In the first stage, PPy nanowires were grown on the surface of GO nanosheet to develop a three-dimensional network with a large number of pores through the layers, which could avoid the re-stacking of GO nanosheet layers. After carbonization, the precursor is transformed into the sandwich-type porous carbon conductive network where the N-doped carbon nanowries (NCNWs) and rGO nanosheets overlapped with each other. The doping of N elements has been demonstrated to result in an augmentation of defects and an increase in the number of active sites [26,27]. In the second stage, through a simple co-precipitation process, the Ni(OH)_2_ nanosheets were uniformly grown on the surface and inside of the NCNWs/rGO substrate.

This structure design allowed for massive loading and exposed the internal active surface, reducing the diffusion distance of the electrolyte ions and increasing the utilization of the internal active sites, thereby improving the electrochemical performance. In addition, the conductive network running through the interior provided more electron transfer paths, which improved conductivity. In order to investigate the effect of this substrate structure on the capacitive performance, we prepared Ni(OH)_2_/rGO and Ni(OH)_2_/NCNWs/rGO with different ratios as a comparative, including Ni_7_/rGO, Ni_5_/NCNWs/rGO, Ni_7_/NCNWs/rGO, and Ni_9_/NCNWs/rGO, with the subscript number representing the load ratio. Through our experiments, it was found that Ni(OH)_2_/NCNWs/rGO not only possessed the maximum C_sp_ (2016.6 F g^−1^, Ni_9_/NCNWs/rGO), but also exhibited higher C_sp_ (931.1 F g^−1^, Ni_5_/NCNWs/rGO) than Ni(OH)_2_/rGO (931.1 F g^−1^, Ni_7_/rGO) with even less loading ratio at the same scan rate of 1 mV s^−1^. These results demonstrated the significant advantage of an NCNWs/rGO substrate compared to a rGO substrate and revealed the effectiveness of this strategy and the excellent potential of the constructed ternary composites as supercapacitors.

## 2. Results and Discussion

### 2.1. Material Characterization

**Figure 1:**
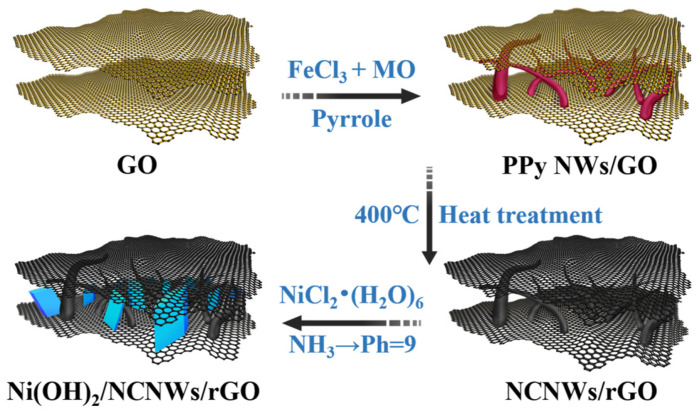
The synthesis procedures of Ni(OH)_2_/NCNWs/rGO.

The preparation process of the composites is illustrated in Figure 1. First, polypyrrole nanowires (PPy NWs) were grown in situ on graphene oxide nanosheets to obtain PPy NWs/GO. Subsequently, the PPy NWs were carbonized to N-doped carbon nanowires (NCNWs) at high temperature, and GO was simultaneously reduced to rGO. Then, an NCNWs/rGO substrate with an interconnected porous structure was obtained. Subsequently, in an alkaline NiCl_2_ solution, Ni(OH)_2_ was generated and co-precipitated with NCNWs/rGO to produce the final product Ni(OH)_2_/NCNWs/rGO. Based on the thermogravimetric analysis (TGA) results (Figure S1), the mass ratio of Ni(OH)_2_ and NCNWs/rGO and were determined to be up to about 9:1 [28], which illustrated an ultra-high loading ratio of Ni(OH)_2_ into NCNWs/rGO.

**Figure 2:**
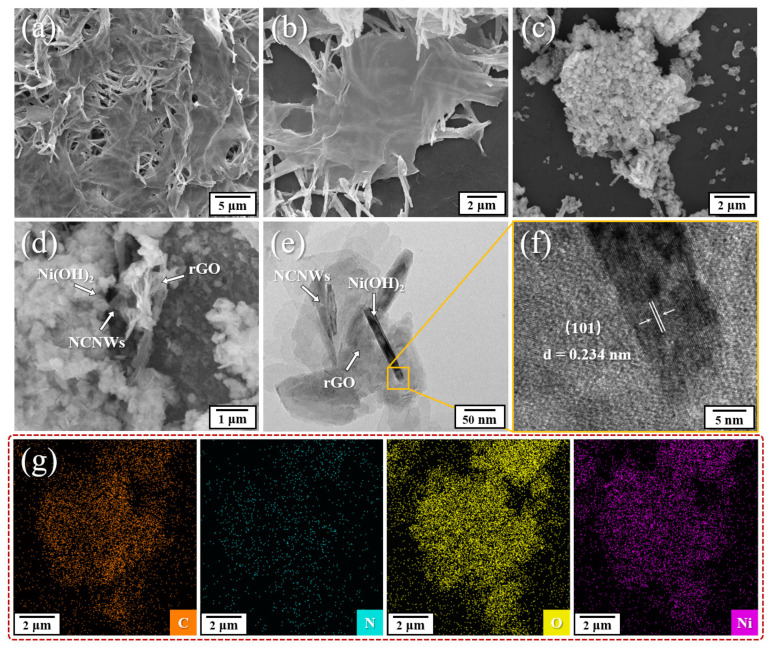
(**a**,**b**) SEM images of PPy NWs/GO and NCNWs/rGO; (**c**,**d**) SEM images Ni(OH)_2_/NCNWs/rGO at different magnifications; (**e**,**f**) HRTEM images of Ni(OH)_2_/NCNWs/rGO at different magnifications; (**g**) EDS mapping images of C, N, O and Ni at the same area as (**c**).

The morphology of all the synthesized samples was characterized using scanning electron microscope (SEM) and transmission electron microscope (TEM). GO nanosheets have a typical two-dimensional microsheet structure (Figure S2), while PPy nanowires (PPy NWs) have a one-dimensional structure and intersect each other to form a three-dimensional network structure (Figure S3). Figure 2a shows that PPy NWs grows uniformly between GO nanosheets, constructing a large number of pores and channels, which forms a sandwich-type structure of nanosheet–nanowire layered overlaps. Notably, in Figure 2b, after high-temperature treatment, the sandwich-type structure was not destroyed and the NCNWs and GO nanosheets remained firmly bonded. Such a structure is expected to provide more loading sites, more electron transport paths and shorter ion-diffusion channels for the inner functional materials. This structure can avoid accumulation of subsequent materials and expose more active surfaces of the electrode material. As shown in Figure 2c,d, Ni(OH)_2_/NCNWs/rGO exhibits a typical crumpled and porous surface. It is the specific way of combining the three components additionally shown in Figure 2d that Ni(OH)_2_ nanosheets are uniformly and massively loaded on the surface and interior of the NCNWs/rGO with a lot of N-doped carbon nanowires inside. In contrast, as depicted in Figure S4, Ni(OH)_2_/rGO presents a blocky morphology, and a large number of densely stacked nanosheets can be observed on the surface. This suggests that the design of the NCNWs/rGO substrate plays an important role in the subsequent loading of the Ni(OH)_2_ materials. A high-resolution TEM image (Figure 2e,f) shows that the lattice spacing is approximately 2.34 Å, corresponding to the (101) crystal plane of Ni(OH)_2_. Furthermore, the thickness of this Ni(OH)_2_ nanosheets crystal is approximately 11.5 nm by measuring the section of the TEM image. As demonstrated in EDS mapping images (Figure 2g), all elements including C, N, O and Ni appear uniformly in the Ni(OH)_2_/NCNWs/rGO, confirming the perfect combination of Ni(OH)_2_, NCNWs, and rGO in the final composite. N elements uniformly distributed throughout the carbon substrate, and the existence of N atoms can induce abundant surface defects in the carbon material, which contribute to provide better electrochemical activity.

**Figure 3:**
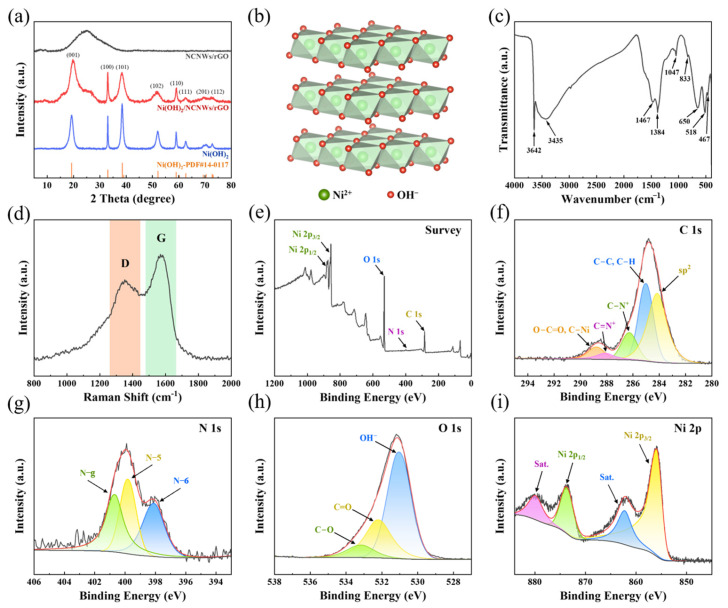
(**a**) XRD patterns of Ni(OH)_2_/NCNWs/rGO, NCNWs/rGO and Ni(OH)_2_; (**b**) crystal structure diagram of Ni(OH)_2_; (**c**) FTIR spectra and (**d**) Raman spectra of Ni(OH)_2_/NCNWs/rGO; (**e**) XPS spectra of Ni(OH)_2_/NCNWs/rGO; (**f**–**i**) high-resolution XPS spectra of (**f**) C 1s, (**g**) N 1s, (**h**) O 1s, and (**i**) Ni 2p of Ni(OH)_2_/NCNWs/rGO; all black and red lines in the subfigures are raw data lines and fitting data lines, respectively.

The crystal structure of the Ni(OH)_2_/NCNWs/rGO samples was confirmed by XRD analysis (Figure 3a). Compared with pure Ni(OH)_2_ and NCNWs/rGO XRD pattern, Ni(OH)_2_/NCNWs/rGO possesses the characteristic diffraction peaks of both materials, which indicates a successful synthesis of the Ni(OH)_2_/NCNWs/rGO composite. The diffraction peaks at 19.76°, 33.04°, 38.45° 52.03°, and 59.15° could be indexed to the crystal plane of (001), (100), (101), (102), and (110) of Ni(OH)_2_ (JCPDS No. PDF#14-0117) [29,30]. The (001) diffraction plane of Ni(OH)_2_ demonstrates a distinct high-angle shift. Bragg’s law analysis at 2θ = 19.76° yields an interplanar spacing of d≈4.50 Å, exhibiting 2.2% lattice contraction compared to the theoretical value (4.60 Å), which confirms compressive lattice strain formation [31]. Additionally, it can be seen that these peaks have broader widths than pure Ni(OH)_2_, indicating that the grain size in the composite material is smaller. A smaller size can expose more active surface areas, improve material utilization, and demonstrate higher electrochemical activity. The crystal structure of Ni(OH)_2_ is illustrated in Figure 3b.

In the FTIR spectrum of the composite material (Figure 3c), the broad peak at 3435 cm^−1^ corresponds to hydrogen-bonded O-H stretching vibrations of Ni(OH)_2_. The sharp peak at 3642 cm^−1^ is attributed to isolated hydroxyl groups or residual N-H bonds in N-doped carbon. The strong peaks at 1467 cm^−1^ and 1384 cm^−1^ arise from C-N vibrations in the nitrogen-doped carbon framework and sp^2^-hybridized C=C stretching modes, respectively. The intense absorption at 650 cm⁻^1^ is unambiguously assigned to Ni-O lattice vibrations, while the peaks at 518 cm^−1^ and 476 cm^−1^ are likely attributed to overlapping signals from Ni-O vibrations and interlayer shear modes of the carbon matrix. Weak peaks at 1086 cm^−1^ and 1047 cm^−1^ indicate trace residual C-O-C groups in reduced graphene oxide [32,33]. These spectral features collectively verify the formation of a composite structure comprising Ni(OH)_2_ nanoparticles and N-doped carbon/graphene, confirming the successful loading of nickel hydroxide. The intense absorption at 3435 cm^−1^ indicates reduced OH⁻ adsorption energy in Ni(OH)_2_/NCNWs/rGO, promoting the adsorption of OH^−^ ions, which is beneficial for enhancing capacitive performance in alkaline electrolytes [34].

To obtain further information of the type of carbon in the Ni(OH)_2_/NCNWs/rGO, Raman spectroscopy was performed in Figure 3d. 1350 cm^−1^ and 1577 cm^−1^ are separately corresponding to D and G bands. The D-band vibration, arising from structural defects in the hexagonal carbon network, manifests aromatic ring distortions, while the G-band corresponds to in-plane vibrational modes of sp^2^ bonded carbon [35]. Notably, the value of I_D_/I_G_ ratios of Ni(OH)_2_/NCNWs/rGO is 0.75, suggesting that there are abundant defects in NCNWs/rGO that are beneficial to improve the electrochemical performance.

We used X-ray photoelectron spectroscopy (XPS) to analyze the elemental composition and electronic states of the prepared samples. The XPS survey of Ni(OH)_2_/NCNWs/rGO (Figure 3e) exhibits the characteristic peaks of C, N, O, and Ni elements. Compared with the curve of PPy NWs/GO (Figure S5), the Ni(OH)_2_/NCNWs/rGO sample exhibit a clear high-intensity response peak of Ni element, indicating the successful synthesis of a composite material. C 1s spectroscopy has a main peak (Figure 3f) located at 248.16 eV due to sp^2^ carbon atoms, showing the typical characteristic of graphene sheets and carbon nanowires [36]. The component situated at 285.0 eV is related to sp^3^ carbon atoms, and it is a proof of abundant defects existing in the reduced graphene oxide structure [37]. The other peaks located at 286.28 eV, 288.07 eV, and 288.78 eV can be attributed to C−N^+^, C=N^+^ O−C=O, and C−Ni, respectively [33,38]. The N 1s peaks (Figure 3g) can be divided into three peaks located at 398.11 eV, 399.81 eV, and 400.67 eV, corresponding to the pyridinic-N (N-6), pyrrolic-N (N-5), and graphitic-N (N-g) [39]. O 1s spectroscopy has a strong main peak (Figure 3h) located at 531.03 eV due to OH^−^ [40], while the other two weak components located at 532.19 eV and 533.15 eV are related to C=O and C−O [41]. In the Ni 2p XPS spectrum (Figure 3i), two main bands corresponding to spin-orbit splitting, with Ni 2p 3/2 located at about 855.91 eV [42] and Ni 2p 1/2 at about 873.68 eV, and two satellite peaks, which are indexed to Ni^2+^ [43].

**Figure 4:**
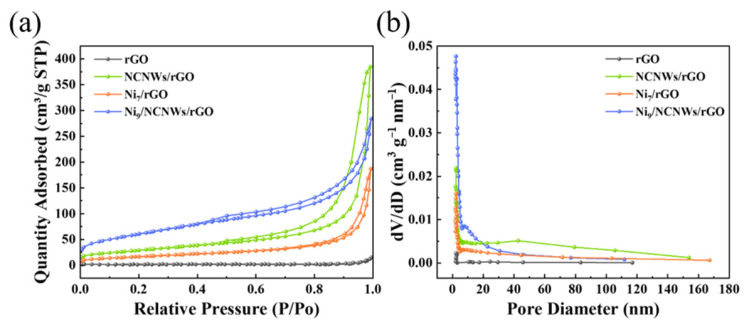
(**a**) The N_2_ adsorption–desorption isotherm curves and corresponding (**b**) pore size distribution curves for rGO, NCNWs/rGO, Ni(OH)_2_/rGO, and Ni(OH)_2_/NCNWs/rGO.

Furthermore, the specific surface area and pore structure of the carbon-containing composite materials can greatly affect the ion adsorption and transport, which in turn affects their capacitive storage performance. The N_2_ adsorption–desorption isotherms in Figure 4a exhibit typical IV characteristics and H3-type hysteresis loops, indicating the presence of a layered porous structure in the other three materials except for rGO nanosheets [44]. As seen in the curve of rGO nanosheets, it should be noted that there are almost no pores or channels, but only some pores created by the stacking of nanosheets. The NCNWs/rGO sample, on the other hand, has a significantly higher specific surface area, demonstrating the porous structure of the carbon nanowires. The specific surface area of the rGO nanosheet, NCNWs/rGO, Ni(OH)_2_/NCNWs/rGO, and Ni(OH)_2_/rGO were found to be 4.1, 107.2, 221.3, and 62.3 m^2^ g^−1^, respectively. The expansion in the specific surface area of the Ni(OH)_2_/NCNWs/rGO materials can be ascribed to the intercalation of functional material, effectively preventing compact stacking of rGO nanosheet layers and exposing more active surface. Compared with Ni(OH)_2_/rGO, this effect can be achieved more efficaciously by introducing N-doped nanowires into rGO nanosheets than Ni(OH)_2_ only. Similarly, the pore size distribution curves (Figure 4b) also show that both NCNWs/rGO and Ni(OH)_2_/NCNWs/rGO have a significantly increased number of pores compared with rGO nanosheets and Ni(OH)_2_/rGO, which is obviously contributed by the insertion of NCNWs. NCNWs is distributed in both macropores and mesopores, while Ni(OH)_2_/NCNWs/rGO is mainly distributed in mesopores, suggesting that the massive growth of C in the pores fills the space inside the substrate material. According to Table 1, these materials exhibit a range of pore sizes from 7 to 23 nm, indicating the coexistence of micropores and mesopores. Contrasting NCNWs/rGO with Ni_9_/NCNWs/rGO, the minification of pore volume (0.59 cm^3^ g^−1^ to 0.37 cm^3^ g^−1^) and pore size (22.1 nm to 7.9 nm) can be attributed to the Ni(OH)_2_ growing in the pores. This can bring higher internal space utilization and higher load capacity. In addition, they provide abundant interfaces, more electrons transport channels, and shorter ions diffusion paths, which accelerate the transport and diffusion of electrolyte ions and enhance the ability to store charge [39].

### 2.2. Electrochemical Performance

**Figure 5:**
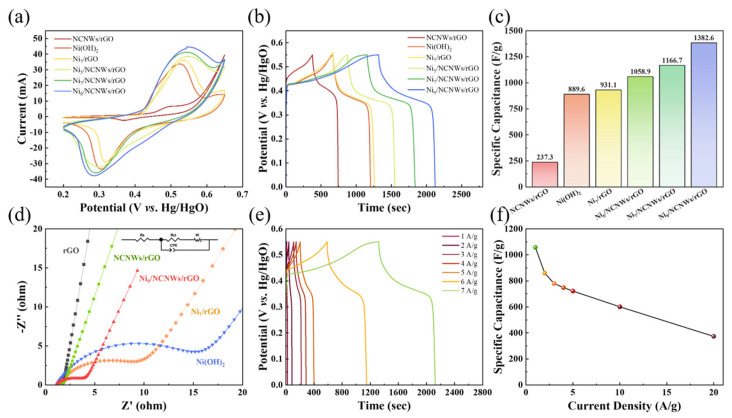
Comparison of electrochemical properties of NCNWs/rGO, Ni(OH)_2_, Ni(OH)_2_/rGO, and Ni(OH)_2_/NCNWs/rGO in 1 M KOH. (**a**) CV curves at a scan rate of 5 mV s^−1^; (**b**) GCD curves at a current density of 1 A g^−1^; (**c**) bar chart of specific capacity values; (**d**) EIS curves; (**e**) GCD curves of Ni(OH)_9_/NCNWs/rGO at different current densities; (**f**) specific capacitance curves of Ni(OH)_9_/NCNWs/rGO at different current densities.

The electrochemical performance of as-synthesized samples of NCNWs/rGO, Ni(OH)_2_, Ni(OH)_2_/rGO, and Ni(OH)_2_/NCNWs/rGO has been evaluated with the three electrodes system. In Figure 5a, the cyclic voltammetry (CV) curves of these samples show a couple of obvious redox peaks, a manifestation of typical pseudocapacitance behavior. This is mainly ascribed to the reaction as following [45]:

(1) $${\mathrm{Ni}(\mathrm{OH})}_{2}+{\mathrm{OH}}^{-}\overset{\;\mathrm{discharge}/\mathrm{charge}\;}{↔}\;\mathrm{NiOOH}+{\;H}_{2}O+{\;e}^{-}$$

At the scan rate of 5 mV s^−1^, the CV curve of Ni_9_/NCNWs/rGO has the largest enclosing area, indicating a significant improvement in capacitance performance compared to other samples. This improvement can be attributed to the optimization of the substrate structure by interposition of NCNWs network and the increasing of loading mass of Ni(OH)_2_; both aspects are beneficial to enhance the electrochemical performance of the electrodes. Figure 5b shows the galvanostatic discharge–charge (GCD) curves at the 0–0.57 V. The Ni_9_/NCNWs/rGO displays the longest discharging time than others, confirming the same trend of specific capacitance found by the CV study. According to Equation (6), their C_sp_ values are listed in Figure 5c. The C_sp_ are 1382.6, 1166.7, 1058.9, 931.1, 889.6, and 237.3 F g^−1^, respectively corresponding to Ni_9_/NCNWs/rGO, Ni_7_/NCNWs/rGO, Ni_5_/NCNWs/rGO, Ni_7_/rGO, NCNWs/rGO. Their specific capacity (Q_sp_) can be calculated by using Equation (8), and their value of Q_sp_ are 172.8, 145.8, 132.4, 116.4, 111.2, and 29.66 mAh g^−1^ in sequence.

To deeply research the internal resistance, the EIS measurement was performed. The Nyquist plots and the equivalent circuit are illustrated in Figure 5d. All EIS curves have a semicircle part and a linear part. The diameter of the semicircle represents the charge–transfer resistance (R_ct_). The horizontal axis intercept represents the equivalent series resistance (R_s_), which is composed of intrinsic resistance, electrolyte resistance, and interface contact resistance. It can be seen that each sample has a similar R_s_ value, indicating that all the samples have lower intrinsic impedance. However, the R_ct_ of Ni_9_/NCNWs/rGO, Ni_7_/rGO, and Ni(OH)_2_ are 2.6 Ω, 9.1 Ω, and 13.3 Ω (Table 2), confirming that the NCNWs/rGO reduce R_ct_ effectively. In the region of low-frequency, the slope of the Ni_9_/NCNWs/rGO increases compared to Ni_7_/rGO and Ni(OH)_2_, standing for less resistance to ions transfer. These improvements collectively demonstrate that Ni_9_/NCNWs/rGO has better favorable capacitive properties and high conductivity. In order to study the rate performance of materials in detail, GCD curves of Ni_9_/NCNWs/rGO at different current densities are tested in Figure 5e. It can be seen that the GCD curves maintain a similar charging and discharging plateau at different current densities. According to Equation (7), the values of C_sp_ at different current densities are calculated in Figure 5f. Specifically, at 1, 2, 3, 4, 5, 10, and 20 A g^−1^, the C_sp_ are 1253.6, 1019.1, 924.3, 886.9, 855.6, 712.2, and 442.4 F g^−1^, and the Q_sp_ are 191.5, 155.7, 141.2, 135.5, 130.7, 108.8, and 67.6 mAh g^−1^, respectively.

**Figure 6:**
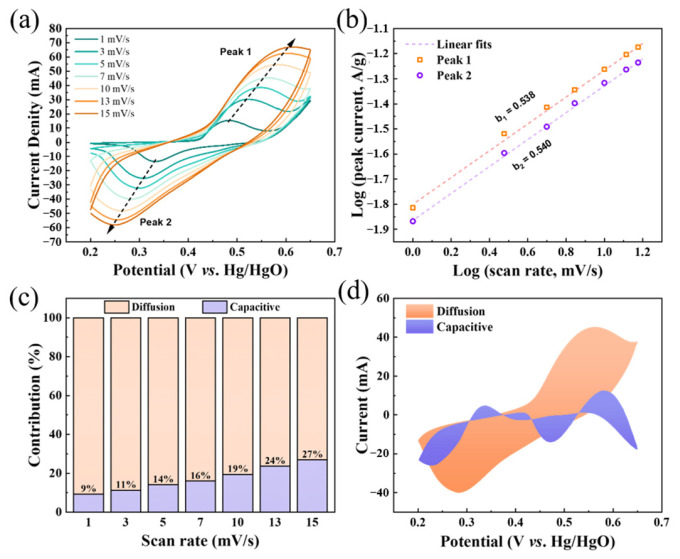
(**a**) CV curves of Ni_9_/NCNWs/rGO at different scan rates; (**b**) logarithmic relationship between the redox peak currents and scan rates; (**c**) capacitance contribution ratios of the Ni_9_/NCNWs/rGO electrode at different scan rates; (**d**) separation of capacitance and diffusion currents at 5 mV s^−1^.

To comprehensively understand the kinetic properties of charge storage of Ni_9_/NCNWs/rGO, it is indispensable to analyze the electrochemical behavior. Figure 6a shows its variation of CV curves with the scan rate range from 1 mV s^−1^ to 15 mV s^−1^. The area of the CV curve gradually enlarges as the scan rate increases. Their C_sp_ are listed in Figure S6, and calculated for 2016.6, 1663.7, 1382.6, 1226.9, 1008.6, 826.8, and 728.3 F g^−1^, respectively corresponding to 1, 3, 5, 7, 10, 13, and 15 mV s^−1^. Besides, the redox peaks shift to a longer distance from each other while always maintaining excellent symmetry, indicating a good reversibility of the electrode. consists of two main mechanisms, capacitance-controlled and diffusion-controlled processes. The charge storage consists of two primary mechanisms, namely capacitance-controlled and diffusion-controlled process. According to CV test, the different process can be distinguished by the following equations [46]:

(2) $$i={a\nu }^{b}$$

(3) log⁡i=log⁡a+νlog⁡b

where *i* (A) is the peak current, *ν* (V/s) is the scan rate, and a and b are variables. The value of *b* can be solved by linear fitting. *b* = 0.5 corresponds to the diffusion-controlled process, and *b* = 1 corresponds to the capacitance-controlled process, while 0.5 ˂ *b* ˂ 1 indicates that diffusion-controlled and capacitance-controlled process coexist. As shown in Figure 6b, both *b*-values are approximately 0.54, indicating that the Ni_9_/NCNWs/rGO has a typical pseudocapacitance characteristic. To further clarify the primary mechanism in the charge storage process of the Ni_9_/NCNWs/rGO, the percentage of the capacitive contribution between diffusion and capacitance process at different scan rate can be calculated by the following equations [46]:

(4) $$i\left(\nu \right)=k_{1}\nu +k_{2}\nu^{1/2}$$

(5) $$i\left(\nu \right)/\nu^{1/2}=k_{1}\nu^{1/2}+k_{2}$$

where k_1_*ν* is the current under capacitance control and k_2_*ν*^1/2^ is the current under diffusion control. As shown in Figure 6c, when the scan rate gradually increases, the proportions of the capacitive contribution and diffusion contribution separately increases and decreases, indicating that the capacitive process at high scan rate is dominated by the surface rapid charge storage and release. The proportion of the capacitive contribution at a scan rate of 5 mV s^−1^ is depicted in Figure 6d. The pseudocapacitance contribution of diffusion is 85.8% of the total capacitance. These results collectively confirm that the Ni_9_/NCNWs/rGO electrode material is competent for the application to establish the supercapacitor.

**Figure 7:**
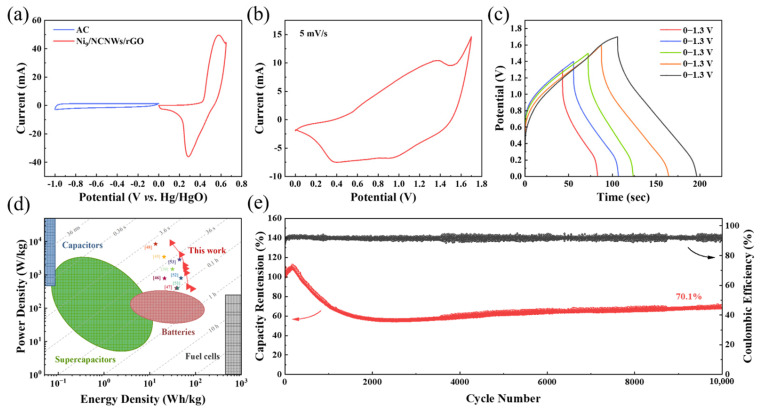
(**a**) CV curves of Ni_9_/NCNWs/rGO and AC at 5 mV s^−1^; (**b**) CV curve of the Ni_9_/NCNWs/rGO//AC asymmetric supercapacitor at a scan rate of 5 mV s^−1^; (**c**) GCD curves of the device at different voltages at a current density of 5 A g^−1^; (**d**) Ragone plots in comparison with other materials reported; (**e**) cycle performance of Ni_9_/NCNWs/rGO//AC asymmetric supercapacitor.

In order to further demonstrate the potential practical applications of Ni_9_/NCNWs/rGO materials, an asymmetric supercapacitor with Ni_9_/NCNWs/rGO as the positive electrode and commercial activated carbon (AC) as the negative electrode was assembled. According to Equation (9), the mass ratio of the AC and Ni_9_/NCNWs/rGO is determined as 5:1. Figure 7a shows the CV curves of Ni_9_/NCNWs/rGO and AC at 5 mV s^−1^. The potential window is 0–0.65 V for Ni_9_/NCNWs/rGO cathode and −1–0 V for AC anode. Therefore, the fabricated asymmetric supercapacitor has an extensive potential window of up to 1.65 V, and the CV curve of Ni_9_/NCNWs/rGO//AC is depicted in Figure 7b. According to the GCD curves in Figure 7c, the discharge time increases are accompanied by the corresponding enhancement of the potential, and the C_sp_ are 184.4, 152.0, 132.5, 117.0, and 95.7 F g^−1^, at the potential windows of 1.7, 1.6, 1.5, 1.4, and 1.3 V, respectively. According to Equations (10) and (11), the energy density and power density are calculated in Ragone plot (Figure 7d). The maximum specific energy density is up to 85.2 Wh kg^−1^ at specific power density of 381 W kg^−1^, which are higher than traditional energy storage devices and other Ni-based and graphene-based supercapacitor materials that have been reported [47,48,49,50,51,52,53,54,55,56]. This suggests that Ni_9_/NCNWs/rGO can supply high power density while maintaining high energy density. The estimation of the cyclic performance was tested under current density of 5 A g^−1^, as depicted in Figure 7e. The specific capacitance of the device experienced a growth during the first 300 cycles, which we considered to be a process of electrochemical activation, and then the capacitance gradually decreased and stabilized after about 2000 cycles. After 10,000 charge/discharge cycles, the Ni_9_/NCNWs/rGO//AC asymmetric supercapacitor retains 70.1% of initial capacitance and a 92.7% coulombic efficiency. In order to further verify the stability of B, the tested electrodes were characterized by XRD and XPS. In Figure S7, the XRD image shows the compression between Ni(OH)_2_/NCNWs/rGO and the electrode after a cycling stability test. After 10,000 cycles test, the crystal structure did not change significantly. Figure S8 shows the XPS image of the same electrode. By comparing it with Ni(OH)_2_/NCNWs/rGO before the test, the electrode maintains the stability of the components. Both results confirm that Ni(OH)_2_/NCNWs/rGO electrode has good stability.

## 3. Materials and Methods

### 3.1. Chemicals and Reagents

Graphite nanoparticles (≥99.9%) were bought from XFNANO (Jiangsu, China); carbon black was bought from Maya Reagent (Zhejiang, China); activated carbon (AC, YP50F) was produced by Kuraray (Osaka, Japan); ferric chloride (FeCl_3_, ≥99%), nickel chloride hexahydrate (NiCl_2_·(H_2_O)_6_, ≥99%), polytetrafluoroethylene preparation (PTFE, 60 wt%), and ammonium hydroxide solution (NH_3_·H_2_O, 28%) were obtained from Macklin (Shanghai, China); pyrrole (C_4_H_5_N, ≥99%) was bought from Meryer (Shanghai, China); potassium permanganate (KMnO_4_, AR), potassium nitrate (KNO_3_, AR), hydrochloric acid (HCl, 35%), sulfuric acid (H_2_SO_4_, 98 wt%), and hydrogen peroxide (H_2_O_2_, 30%) were provided from Sinopharm Chemical Reagent Co.; Ltd (Shanghai, China). Nickel foam with a thickness of 1.5 mm was from Kunshan Suntech New Materials Co., Ltd (Kunshan, China). All reagents were used as received, without further purification, in the experiments.

### 3.2. Synthesis

#### 3.2.1. Synthesis of Graphite Oxide (GO)

Graphite oxide was prepared by a modified Hummers method. Sulfuric acid (400 mL, 98 wt%) was added into a 1000 mL beaker, and the beaker was cooled to 0 °C in an ice bath with magnetic stirring. Graphite powder (5 g) and KNO_3_ (5 g) were added to the beaker. Subsequently, 40 g KMnO_4_ was then added slowly in batches, and the whole process lasted for 40 min. The beaker was transferred to a thermostatic oil bath at a constant 38 °C, and was stirred continuously for 4.5 h. Then, it was put in an ice bath with magnetic stirring again. 400 mL deionized water was gradually added portion by portion. The reaction vessel was placed in an oil bath whereupon the temperature rapidly increased to 80 °C, which was maintained for 30 min. The heat was removed and the reaction was cooled to room temperature. The solution was further treated with H_2_O_2_ (40 mL, 30 wt%). The precipitates were washed several times with 5% HCL after centrifugation, then it was transferred into a dialysis tube to be dialyzed with deionized water until pH is 7. After freeze-drying, GO powder was obtained.

#### 3.2.2. Synthesis of PPy NWs/GO Precursor

The GO dispersion was obtained by adding 0.2 g GO powder into 30 mL of deionized water by ultrasonication for 2 h with a bath sonicator. 0.15 mmol (0.0491 g) of methyl orange (MO) was dissolved in 30 mL of deionized water. Subsequently, 0.15 mmol (0.0243 g) of FeCl_3_ was added into the MO solution, and the mixture was subjected to continuous stirring for 20 min. 105 μL (0.1 g) of pyrrole was then injected into the MO-FeCl_3_ solution, and the mixture was stirred for a further 3 h. Thereafter, the GO dispersion was added into the above solution, and the mixture was stirred for a further 9 h. The product was washed with deionized water and ethanol several times. Following this, the PPy NWs/GO sample was obtained after freeze-drying.

#### 3.2.3. Synthesis of NCNWs/rGO and rGO

0.1 g of PPy NWs/GO powder was heated to 400 °C for 1 h in the tube furnace, and the obtained sample was named NCNWs/rGO. The heating rate was 5 °C/min, and N₂ was used as the protective gas. The pure rGO samples were prepared in a similar procedure.

#### 3.2.4. Synthesis of Ni(OH)_2_/NCNWs/rGO, Ni(OH)_2_/rGO and Ni(OH)_2_

The Ni(OH)_2_/NCNWs/rGO composites were synthesized by co-precipitation of Ni(OH)_2_ and NCNWs/rGO. 20 mg of NCNWs/rGO was dispersed into 32 mL of deionized water by ultrasonication for 45 min with a bath sonicator. Thereafter, a calculated amount of NiCl_2_·(H_2_O)_6_ was dissolved in this dispersion and tempestuously stirred for 30 min. 30% NH_3_·H_2_O was then added dropwise until the pH was adjusted to 9. Subsequently, the stirring was stopped, and the mixture was left to age for 12 h. The obtained Ni(OH)_2_/NCNWs/rGO composites were filtered and washed several times with deionized water, and dried at 70 °C for 12 h. According to the weight ratio of Ni(OH)_2_ to NCNWs/rGO, the resulting Ni(OH)_2_/NCNWs/rGO composites were marked as Ni_9_/NCNWs/rGO, Ni_7_/NCNWs/rGO, and Ni_5_/NCNWs/rGO. The synthesis routes are illustrated in Figure 1.

For comparison, the Ni(OH)_2_/rGO sample without NCNWs were prepared by the same process, and the final product was marked as Ni_7_/rGO. Similarly, pure Ni(OH)_2_ nanosheets were also synthesized Ni(OH)_2_ by the same procedure without using any NCNWs or rGO substrate.

### 3.3. Materials Characterization

The morphologies and compositions of the samples were investigated by field-emission scanning electron microscope (SEM, FIB-SEM GX4, Waltham, MA, USA) and transmission electron microscopy (TEM, FEI Tecnai G2 F20, Waltham, MA, USA). The powder X-ray diffraction (PXRD) was tested on SmartLab 9Kw (Tokyo, Japan) with Cu *K*α radiation (*λ* = 1.5418 Å). The chemical states of the samples were detected by X-ray photoelectron spectroscopy (XPS, Escalab Xi+, Waltham, MA, USA). Specific surface area and pore structure were measured by Nitrogen adsorption/desorption (BET, ASAP-2460, Norcross, GA, USA).

### 3.4. Electrochemical Measurement

The electrode material was prepared by mixing active material with carbon black and PTFE binder in anhydrous ethanol at a ratio of 8:1:1. The slurry mixture was then coated onto Ni foams, and the electrodes are then dried at 70 °C. The mass loading of the active materials ranged from 2.0 to 2.2 mg.

The electrochemical measurements including cyclic voltammetry (CV), galvanostatic charge-discharge (GCD), and electrochemical impedance spectroscopy (EIS) of the prepared electrodes were conducted at a CHI 660E (Chenhua, Shanghai) electrochemical workstation. Three electrodes system test: the prepared electrodes were designated as working electrodes, the Pt plate electrode as the counter electrode, and Hg/HgO electrode as the reference electrode. 1 M KOH solution is used as the electrolyte. The specific capacitance (*C_sp_*, F g^−1^) and corresponding specific capacity (*Q_sp_*, mAh g^−1^) can be evaluated by CV and GCD according to the following equations [57,58]:

(6) $$C_{\mathrm{sp}}=\frac{\int \mathrm{IdV}}{m\times ∆V\times \nu }$$

(7) $$C_{\mathrm{sp}}=\frac{2I\int \mathrm{Vdt}}{m\times ({∆V)}^{2}}$$

(8) $$Q_{\mathrm{sp}}=\frac{C_{\mathrm{sp}}\times ∆V}{3.6}$$

where *I* (A g^−1^) is the current, Δ*V* (*V*) is the potential window, *m* (g) is the loading mass, *ν* (mV s^−1^) is the scan rate, *m* (g) is the loading mass of active material, *t* (s) is the discharge time, and  ∫Vdt is the integral of the discharge current with respect to voltage based on GCD plot.

Two electrodes test system: the asymmetric capacitor consists of the prepared electrodes as cathodes, active carbon (AC) as anode, and 1 M KOH as electrolyte. The mass ratio of the anode to cathode can be calculated using the following equation [16]:

(9) $$\frac{m_{+}}{m_{-}}=\frac{C_{-}\times {∆V}_{-}}{C_{+}\times {∆V}_{-}}$$

where *m*_+_ and *m*_–_ are the loading mass (g), *C*_+_ and *C*_–_ are the specific capacitance (F g^−1^), Δ*V*_+_ and Δ*V*_−_ are the voltage range (V), and the plus and minus signs are the cathode and anode electrodes, respectively.

The energy density and power density of supercapacitor devices were concluded by Equation (10) and (11):

(10) $$E=\frac{1}{2}\times C_{\mathrm{sp}}\times {∆V}^{2}$$

(11) $$P=\frac{E}{t}$$

where *E* is the specific energy density (Wh kg^−1^), *P* is the specific power density (W kg^−1^), *C_sp_* is the specific capacitance, ∆*V* is the work voltage, and *t* is the discharge time.

The cycling stability of the asymmetric capacitor was assessed using the Land battery test system (CT 2001 A) with a current density of 1 A g^−1^ at 25 °C.

## 4. Conclusions

In conclusion, we constructed a novel carbon conductive substrate (NCNWs/rGO) using a strategy of combining polypyrrole (PPy) and graphene oxide (GO), followed by carbonization of PPy and reduction of GO. Then, the Ni(OH)_2_/NCNWs/rGO active materials were synthesized by a simple co-precipitation method. A series of morphological and structural characterizations have demonstrated that the NCNWs/rGO substrate not only provides higher specific surface area and suitable pore size distribution, but more importantly, can inhibit Ni(OH)_2_ aggregation and expose more active sites. Therefore, when applied as a cathode material for supercapacitors, the Ni(OH)_2_/NCNWs/rGO exhibits significantly lower impedance, higher specific capacity, and long cycle stability. In the comparison at 5 mV s^−1^, Ni(OH)_2_/NCNWs/rGO shows the highest C_sp_ (1382.6 F g^−1^), greatly exceeding that of Ni(OH)_2_/rGO (931.1 F g^−1^). And its C_sp_ reaches a remarkable specific capacitance of 2016.6 F g^−1^ at 1 mV s^−1^ upon further research. The assembly of asymmetric supercapacitors with activated carbon anode also demonstrates its potential for practical applications. The asymmetric supercapacitor of Ni(OH)_2_/NCNWs/rGO//AC can work at a high voltage range of 0–1.65 V, and has a maximum specific energy density of 85.2 Wh kg^−1^ at a specific power density of 381 W kg^−1^. Furthermore, the asymmetric supercapacitor remained 70.1% capacitance retention after 10,000 cycles and kept a 92.7% coulombic efficiency. These findings reveal the possible prospect of nickel hydroxide, carbon nanowires, and graphene-based asymmetric supercapacitors in high power- and high energy-required applications.

## Figures and Tables

**Table 1:** Specific surface areas and pore parameters of rGO, NCNWs/rGO, Ni_7_/rGO, and Ni_9_/NCNWs/rGO.

Sample	Specific Surface Area ^1^ (m^2^ g^−1^)	Pore Volume ^2^ (cm^3^ g^−1^)	Average Pore Size (nm)
rGO	4.1	0.02	22.8
NCNWs/rGO	107.2	0.59	22.1
Ni_9_/NCNWs/rGO	221.3	0.37	7.9
Ni_7_/rGO	62.3	0.29	18.6

^1^ Specific surface area (S_BET_) was calculated using the Brunauer–Emmett–Teller (BET) method. ^2^ The total pore volume was calculated the BJH Adsorption Cumulative Pore Volume (BPV) method.

**Table 2:** The R_ct_ of rGO, NCNWs/rGO, Ni_9_/NCNWs/rGO, Ni_7_/rGO, Ni(OH)_2_ electrodes.

**Samples**	rGO	NCNWs/rGO	Ni_9_/NCNWs/rGO	Ni_7_/rGO	Ni(OH)_2_
**R_ct_ (Ω)**	0.26	0.78	2.64	9.13	13.29

## Data Availability

Data will be made available on request.

## Supplementary Materials

The following supporting information can be downloaded at: https://www.mdpi.com/article/10.3390/molecules30051119/s1, Table S1: title The Csp of reported materials consisting of graphene, PANI, N-CNF, g-C_3_N_4_ and Ni(OH)_2_; Figure S1: TGA curve of Ni_9_/NCNWs/rGO; Figure S2: SEM image of rGO nanosheets; Figure S3: SEM image of PPy NWs; Figure S4: SEM image of Ni(OH)_2_/rGO; Figure S5: XPS spectra of PPy NWs/GO before high temperature treatment; Figure S6: Separation of specific capacitance at different scan rate; Figure S7: XRD image of the compression between Ni(OH)_2_/NCNWs/rGO and the electrode after cycling stability test; Figure S8: High-resolution XPS spectra of the Ni(OH)_2_/NCNWs/rGO electrode after cycling stability test. (a) C 1s, (b) Ni 2p. References [20,21,22,23,24,25,28] are cited in the Supplementary Materials as well.
