# Supplementary material for: Facile Synthesis of Sandwich-Type Porous Structured Ni(OH)2/NCNWs/rGO Composite for High Performance Supercapacitor
Source: Molecules. 2025 Feb 28;30(5):1119. doi: 10.3390/molecules30051119 (PMC11901611; doi:10.3390/molecules30051119)
Supplement: Supplementary file 1 [file molecules-30-01119-s001.zip › molecules-3483684-supplementary.pdf]

Supplementary File For

Facile synthesis of sandwich-type porous structured $Ni(OH)_2$/NCNWs/rGO composite for high performance supercapacitor

Xiaosen Duan [1], Mingyu Dou [1], Lingyang Liu [1,*], Long Zhang [1], Xianrui Bai [1], Ruixin Yang [1], Hengyi Wang [1] and Jianmin Dou [1,*]

[1] Shandong Provincial Key Laboratory of Chemical Energy Storage and Novel Cell Technology, School of Chemistry and Chemical Engineering, Liaocheng University, Liaocheng 252059, China; e-mail@e-mail.com

* Correspondence: liulingy0425@163.com or liulingyang@lcu.edu.cn (L. L.); jmdou@lcu.edu.cn (J. D.)

Table S1 The C_{sp} of reported materials consisting of graphene, PANI, N-CNF, g-C_3N_4 and $Ni(OH)_2$.

Electrode matertials	Preparation method	C_{sp} (F/g)	Electrolyte	Test Condition	Ref.
EGM/rGO	Self-assembly	231	$EMIMBF_4$	1 A/g	[1]
PANI/rGO	Hydrothermal	854	1 M H_2SO_4	1 A/g	[2]
β-$Ni(OH)_2$/rGO	Hydrothermal	802	3 M KOH	2 A/g	[3]
$Ni(OH)_2$/N-CNF	Coprecipitation	1045	6 M KOH	1 mV/s	[4]
$Ni(OH)_2$/HGO	Hydrothermal	1431	6 M KOH	5 A/g	[5]
$Ni(OH)_2$/g-C_3N_4/rGO	Hydrothermal	1785	6 M KOH	2 A/g	[6]

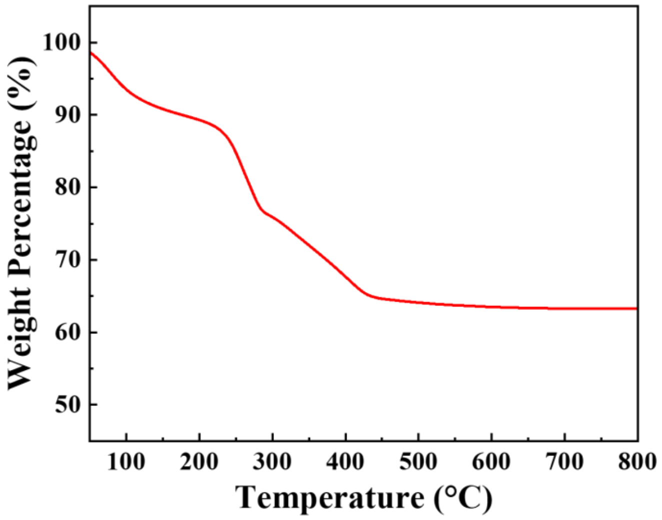

Figure S1. TGA curve of Ni_9/NCNWs/rGO.

The thermogravimetric analysis (TGA) was performed for the Ni_9/NCNWs/rGO powders to find the weight percentage of $Ni(OH)_2$ and NCNWs/rGO in pure O_2. As shown in Figure S1, three weight loss regions can be observed. There is a first weight loss under 250 ˚C where the structurally bonded water of $Ni(OH)_2$ is removed, and the weight loss is about 13.1%. Another weight loss is between 250 ˚C and continues to 300 ˚C due to the reaction of NCNWs/rGO and O_2, about 9.9% for weight loss. The third region is between 300 ˚C to 450 ˚C, where the $Ni(OH)_2$ decomposes to NiO, about 17.5% for weight loss. The curve of Ni_9/NCNWs/rGO almost does not change after 450 ˚C showing that NCNWs/rGO in the Ni_9/NCNWs/rGO has been fully burned. The TG analysis shows that the homemade nickel hydroxide has adsorbed/intercalated H_2O molecules, which is in agreement with the results of FTIR (**Figure 3c**). The presence

of H_2O molecules could enhance the electrochemical performance by facilitating proton diffusion through interlayer molecular channels, owing to their function as ionic transport pathways [7]. The solid composite contains 9.9 wt% NCNWs/rGO, with the remaining 91 wt% related to $Ni(OH)_2$ crystallites. Therefore, the mass ratio of $Ni(OH)_2$ and NCNWs/rGO in the Ni_9/NCNWs/rGO is determined to be 9:1

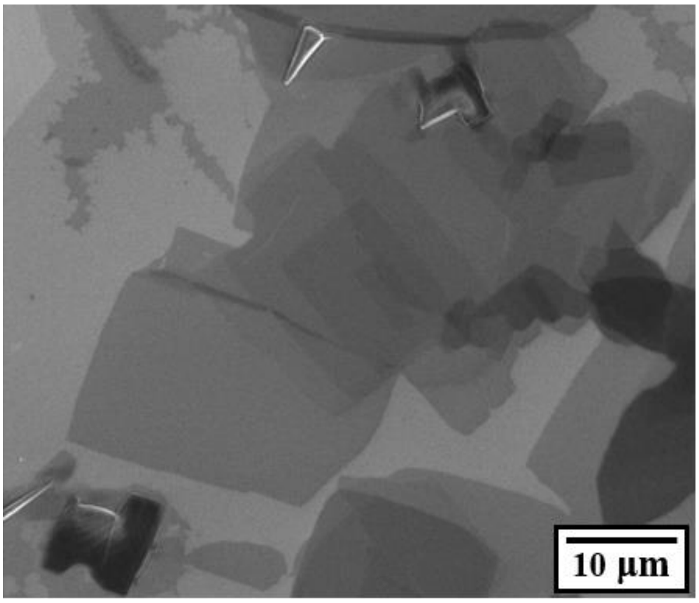

Figure S2. SEM image of rGO nanosheets.

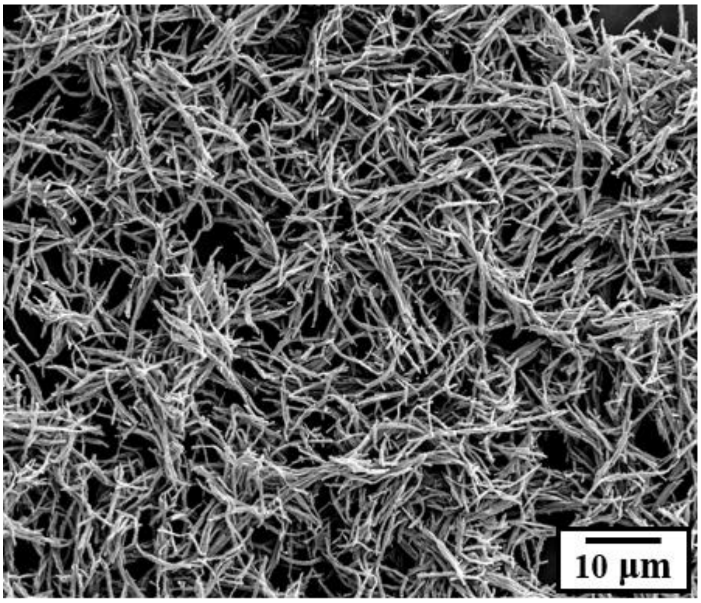

Figure S3. SEM image of PPy NWs.

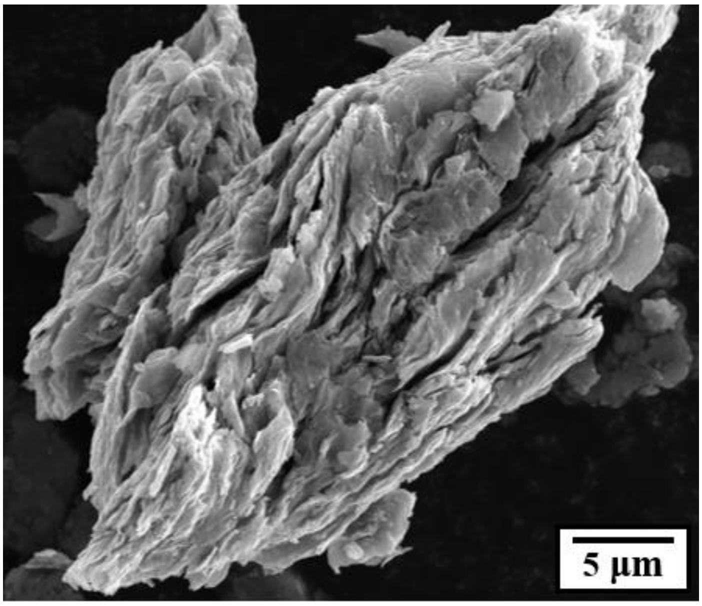

Figure S4. SEM image of $Ni(OH)_2$/rGO

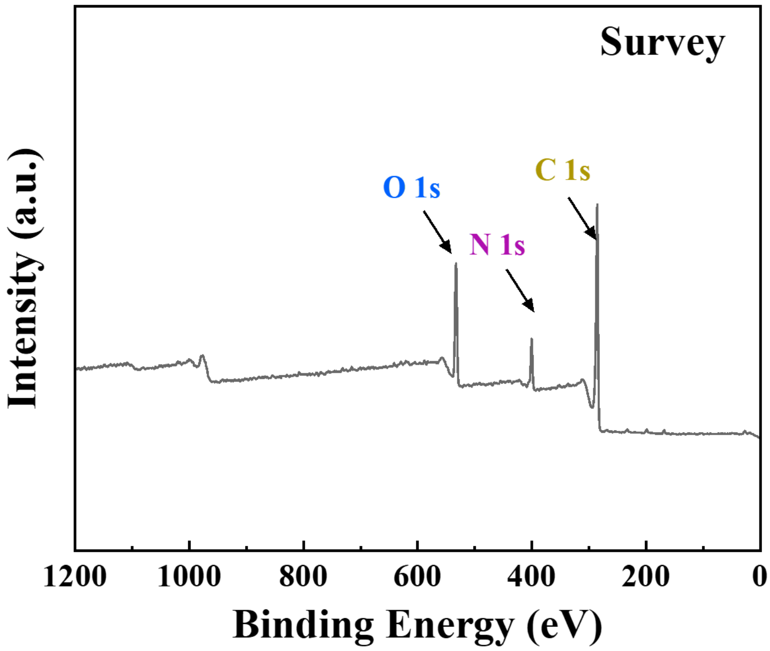

Figure. S5 XPS spectra of PPy NWs/GO before high temperature treatment.

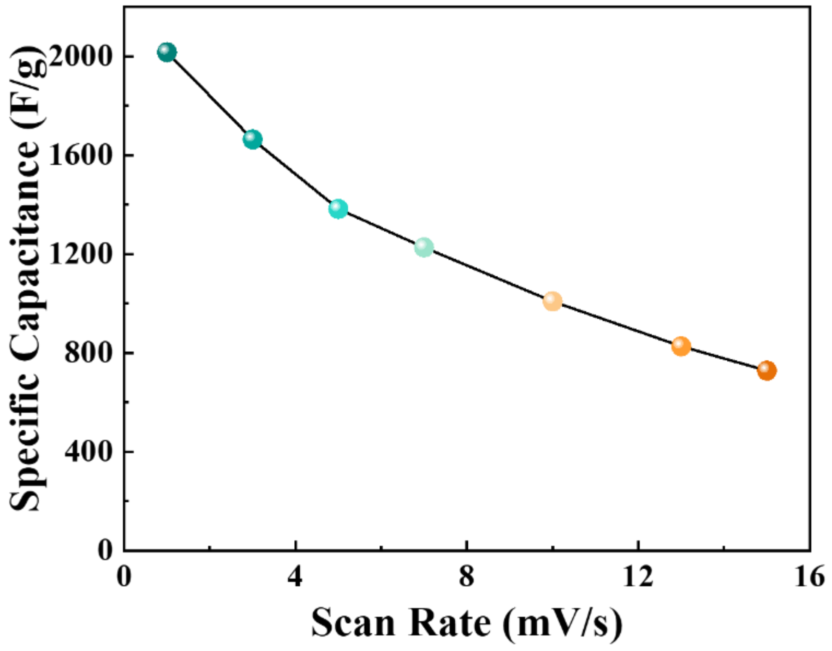

Figure S6. specific capacitance at different scan rate.

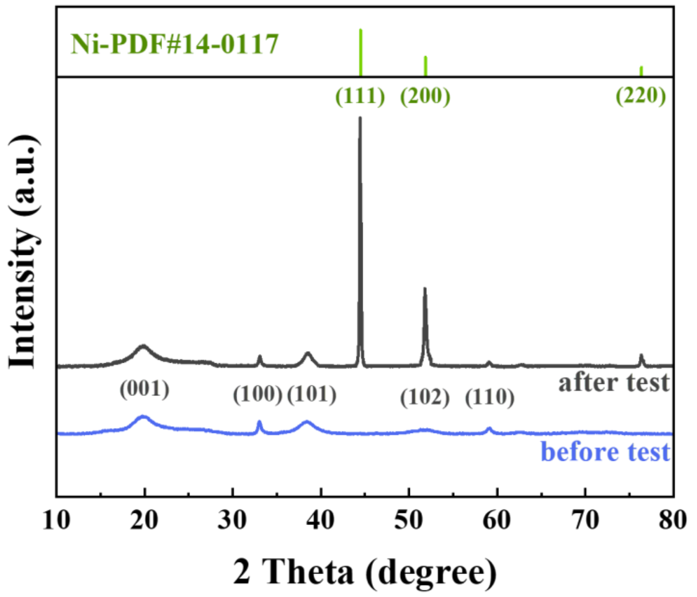

Figure S7. XRD image of the compression between $Ni(OH)_2$/NCNWs/rGO and the electrode after cycling stability test.

As shown in Figure S7, after 10000 cycles test, the diffraction peaks at 19.86°, 33.06°, 38.51° and 59.07° could be indexed to the crystal plane of (001), (100), (101) and (110) of $Ni(OH)_2$ (JCPDS

No. PDF#14-0117), and peaks at 44.44°, 76.34° are belong to the crystal plane of (111) and (220) of nickel (JCPDS No. PDF#04-0850). The result confirmed the good cycling stability of $Ni(OH)_2$/NCNWs/rGO.

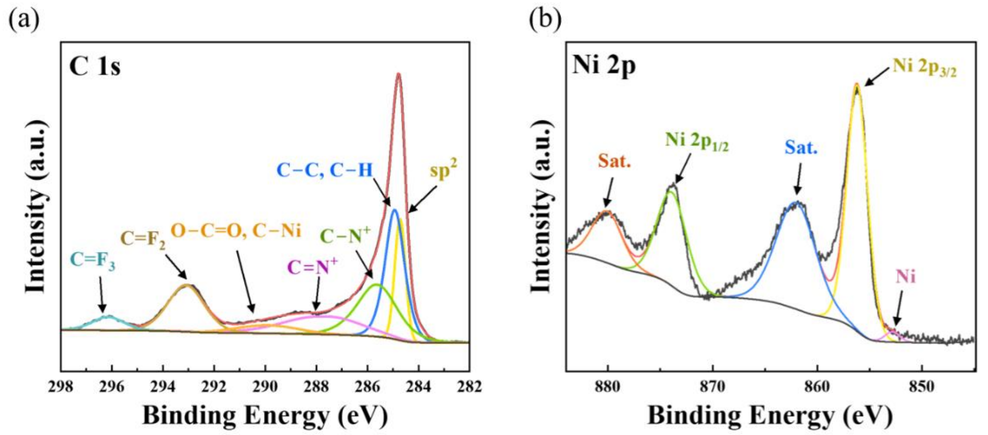

Figure S8. High-resolution XPS spectra of the $Ni(OH)_2$/NCNWs/rGO electrode after cycling stability test. (a) C 1s, (b) Ni 2p.

As shown in Figure S8a, the peak located at 284.7 eV is due to sp_2 carbon, and the peak at 284.95 is belong to sp_3 carbon atoms. The three peaks located at 285.65 eV, 287.7 eV and 289.95 eV can be attributed to C−N+, C=N+ O−C=O and C−Ni, respectively. The other two peaks at 293.1 eV and 296.1 eV are respectively related to $C=F_2$ and $C=F_3$, both due to the addition of PTFE during the preparation of the pole sheet. In Figure S7b two main bands corresponding to spin-orbit splitting, Ni $2p_{3/2}$ is located at about 856.25 eV, Ni $2p_{1/2}$ at about 874.05 eV and two satellite peaks, which are indexed to Ni^{2+}. The peak located at 852.85 eV is due to metal nickel.

References

1. Li, Z.; Gadipelli, S.; Li, H.; Howard, C.A.; Brett, D.J.L.; Shearing, P.R.; Guo, Z.; Parkin, I.P.; Li, F. Tuning the Interlayer Spacing of Graphene Laminate Films for Efficient Pore Utilization towards Compact Capacitive Energy Storage. *Nature Energy* **2020**, *5*, 160–168, doi:10.1038/s41560-020-0560-6.
2. Liu, Z.; Zhao, Z.; Xu, A.; Li, W.; Qin, Y. Facile Preparation of Graphene/Polyaniline Composite Hydrogel Film by Electrodeposition for Binder-Free All-Solid-State Supercapacitor. *Journal of Alloys and Compounds* **2021**, *875*, 159931, doi:10.1016/j.jallcom.2021.159931.
3. Liu, J.; Wang, Y.; Hu, R.; Munir, H.A.; Liu, H. High-Performance Supercapacitor

Electrode Based on 3D Rose-like β-$Ni(OH)_2$/rGO Nanohybrid. *Journal of Physics and Chemistry of Solids* **2020**, *138*, 109297, doi:10.1016/j.jpcs.2019.109297.

4. Cai, J.; Niu, H.; Li, Z.; Du, Y.; Cizek, P.; Xie, Z.; Xiong, H.; Lin, T. High-Performance Supercapacitor Electrode Materials from Cellulose-Derived Carbon Nanofibers. *ACS Applied Materials & Interfaces* **2015**, *7*, 14946–14953, doi:10.1021/acsami.5b03757.
5. Wu, X.; Zeng, F.; Song, X.; Sha, X.; Zhou, H.; Zhang, X.; Liu, Z.; Yu, M.; Jiang, C. In-Situ Growth of $Ni(OH)_2$ Nanoplates on Highly Oxidized Graphene for All-Solid-State Flexible Supercapacitors. *Chemical Engineering Journal* **2023**, *456*, 140947, doi:10.1016/j.cej.2022.140947.
6. Li, L.; Qin, J.; Bi, H.; Gai, S.; He, F.; Gao, P.; Dai, Y.; Zhang, X.; Yang, D.; Yang, P. Ni(OH)2 Nanosheets Grown on Porous Hybrid g-C_3N_4/RGO Network as High Performance Supercapacitor Electrode. *Scientific Reports* **2017**, *7*, 43413, doi:10.1038/srep43413.
7. Liu, C.; Li, Y. Synthesis and Characterization of Amorphous α-Nickel Hydroxide. *Journal of Alloys and Compounds* **2009**, *478*, 415–418, doi:10.1016/j.jallcom.2008.11.049.
